# Supplementary figures and images for: Catenin signaling controls phrenic motor neuron development and function during a narrow temporal window
Source: Front Neural Circuits. 2023 Feb 21;17:1121049. doi: 10.3389/fncir.2023.1121049 (PMC9988953; doi:10.3389/fncir.2023.1121049)

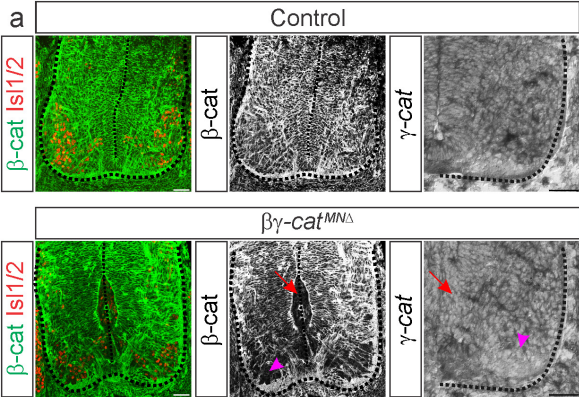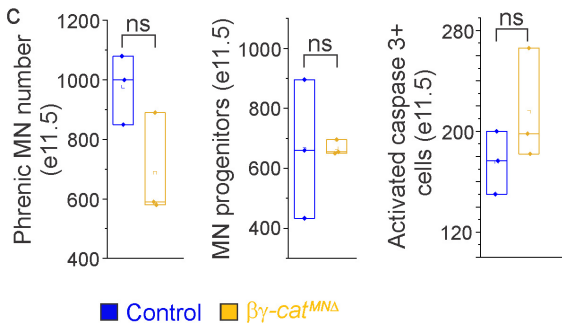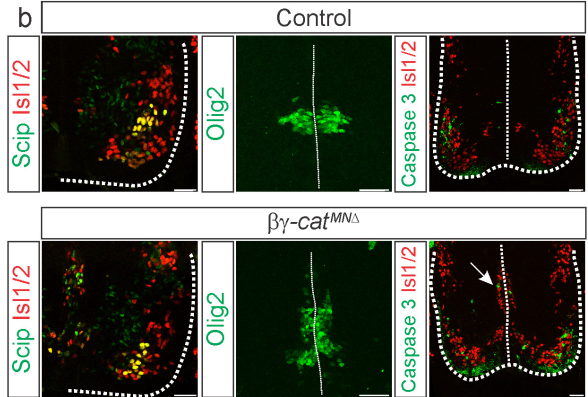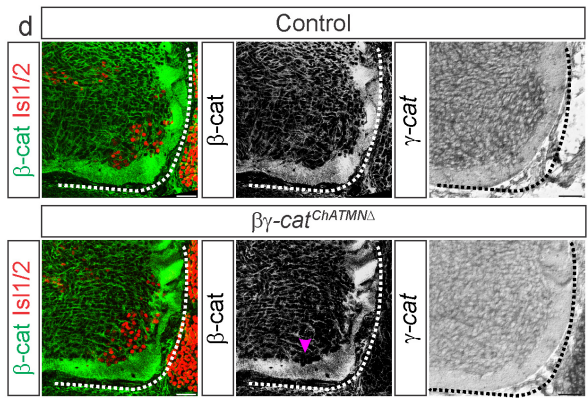

Supplement: Supplementary Figure 1 — Temporally controlled MN-specific catenin inactivation. (a) Olig2::Cre-mediated β- and γ-catenin inactivation results in downregulation of β- and γ-catenin in both MN progenitors (arrow) and MNs (arrowhead) at e11.5. β-catenin protein is visualized by antibody staining (green) while γ-catenin RNA by in situ hybridization (positive signal in black). MNs are labeled by Isl1/2 expression (red). (b) MN progenitor and phrenic MN distribution, but not numbers, are changed in βγ-catMNΔ mice at e11.5. MN progenitors are labeled by the expression of the TF Olig2, while phrenic MNs are labeled by the co-expression of Scip and Isl1/2. Levels of activated caspase 3 (green, right panels) do not significantly change in βγ-catMNΔ mice, although displaced cells close to the midline frequently appear to undergo apoptosis (arrow). (c) Quantitation of MN progenitors, phrenic MNs and apoptotic (caspase 3+) cells in βγ-catMNΔ mice at e11.5. Phrenic MNs were counted as the total number of Scip/Isl1/2+ cells at cervical levels of the spinal cord. MN progenitors and apoptotic cells were counted as the average number of Olig2+ and caspase 3+ cells, respectively, spanning 160 μm of the cervical spinal cord. (d) ChAT::Cre-mediated β- and γ-catenin inactivation results in downregulation of β- and γ-catenin in MNs (arrowhead) at e13.5. Scale bar = 50 μm. [file Image_1.pdf]
